# *Bacillus thuringiensis* bioinsecticide influences *Drosophila* oviposition decision

**DOI:** 10.1098/rsos.230565

**Published:** 2023-08-30

**Authors:** Aurélie Babin, Jean-Luc Gatti, Marylène Poirié

**Affiliations:** Université Côte d'Azur, INRAE, CNRS, Sophia Agrobiotech Institute (ISA), 06903 Sophia Antipolis, France

**Keywords:** *Drosophila*, *Bacillus thuringiensis*, bioinsecticide, behaviour, oviposition

## Abstract

Behavioural avoidance has obvious benefits for animals facing environmental stressors such as pathogen-contaminated foods. Most current bioinsecticides are based on the environmental and opportunistic bacterium *Bacillus thuringiensis* (*Bt*) that kills targeted insect pests upon ingestion. While food and oviposition avoidance of *Bt* bioinsecticide by targeted insect species was reported, this remained to be addressed in non-target organisms, especially those affected by chronic exposure to *Bt* bioinsecticide such as *Drosophila* species. Here, using a two-choice oviposition test, we showed that female flies of three *Drosophila* species (four strains of *D. melanogaster*, *D. busckii* and *D. suzukii*) avoided laying eggs in the presence of *Bt* var. *kurstaki* bioinsecticide, with potential benefits for the offspring and female's fitness. Avoidance occurred rapidly, regardless of the fraction of the bioinsecticide suspension (spores and toxin crystals versus soluble toxins/compounds) and independently of the female motivation for egg laying. Our results suggest that, in addition to recent findings of developmental and physiological alterations upon chronic exposure to non-target *Drosophila*, this bioinsecticide may modify the competitive interactions between *Drosophila* species in treated areas and the interactions with their associated natural enemies.

## 1. Introduction

When exposed to environmental stressors, animals face two main options: dealing with the stressor, which may ultimately lead to the evolution of special features, or physically avoiding it. In the interactions with opportunistic pathogens, broad-sense immunity includes components for dealing with infections (physical barriers and cellular and humoral effectors of the immune system) as well as a behavioural component to physically avoid pathogens and reduce the infection risk [1–3]. The immune response being costly (energy, nutrients and immunopathology resulting from damage to host tissues by effectors of its innate immune response) [2,4], obvious benefits come from physically avoiding pathogens.

Behavioural avoidance of toxic compounds and microorganisms in a foraging context is well documented. Both innate avoidance (disgust) and learned avoidance based on associative learning of hazardous food, are commonly expressed by vertebrates [5] and invertebrates, mainly insects [6–8]. For instance, phytophagous insects avoid plants that accumulate toxic alkaloids [9] and the nematode *Caenorhabditis elegans* prefers feeding on non-pathogenic bacteria over pathogenic ones [10,11]. Exposed to opportunistic pathogens through their diet of overripe fruits, *Drosophila melanogaster* females are able to learn to adjust their preference for a food odour when that odour has previously been associated with the gut infection by the virulent bacterium *Pseudomonas entomophila* [12], as do *C. elegans* nematodes when exposed to pathogenic bacteria [13]. *Drosophila melanogaster* males and females also express strong innate aversive responses to bacterial lipopolysaccharides when feeding and egg laying, respectively, mediated by dTRPA1 cation channels of gustatory neurons [14].

Naturally ubiquitous in the environment, *Bacillus thuringiensis* (*Bt*) is an opportunistic Gram-positive bacterium, which synthesizes insecticidal toxins including Cry proteins as crystals along with spores [15,16]. The insecticidal action relies on the organisms' feeding activity on *Bt*-contaminated food sources [17]. In the context of the growing global food demand and the need for safer and more specific insect pest control, these natural insecticidal properties have led the development of *Bt-*based bioinsecticides (products made of viable *Bt* spores and toxin crystals) or *Bt* transgenic crops to control insect pests in agriculture and forestry (mainly Lepidoptera, and mosquitoes and black flies (Diptera)) [18,19]. Many studies concluded that *Bt* bioinsecticides and *Bt* crops are harmless or have limited impacts on the non-target fauna [20,21]. However, the partial targeting specificity of Cry toxins and the potential for environmental accumulation of spores and toxins upon repeated treatments have raised concern about potential side-effects on non-target organisms [16,22–25]. In insects, recent studies have reported deleterious effects of the Lepidoptera-targeting *Bt* var. *kurstaki* (*Btk*) bioinsecticide on several species of non-target *Drosophila* flies probably present in *Btk*-treated areas. Chronic exposure of fly larvae to subacute doses through the diet altered their growth, development duration, survival and complete development success [26–29]. *Btk* bioinsecticide also impacted the larval metabolism and midgut physiology, impairing protein digestion and disturbing the gut epithelium organization [28]. One way for non-target insects that would alleviate *Bt* bioinsecticide impacts is the expression of behavioural avoidance of *Bt*-treated substrates. As *Bt* bioinsecticides act after ingestion, behavioural avoidance would be advantageous upon food foraging, but also upon female oviposition with direct benefits for the developing offspring and indirect benefits for the female's fitness.

So far, *Bt* behavioural avoidance has been investigated mainly in *Bt*-target invertebrates but scarcely in non-target invertebrates. Studies have reported no change in the oviposition behaviour of *Culex* mosquitoes exposed to *Bt* var. *israelensis* [30] or in the feeding behaviour of the Western corn rootworm *Diabrotica virgifera virgifera* [31], and even an attractive effect of *Bt* maize on the oviposition of the fall armyworm *Spodoptera frugiperda* [32]. Conversely, behavioural avoidance of *Bt* upon food foraging was reported in the nematode *Caenorhabditis elegans* [33–36] and in two Lepidopteran pests, the cotton bollworm *Helicoverpa armigera* and the cotton leafworm *Spodoptera litura* [37]. Females of *H. armigera* and the diamondback moth *Plutella xylostella* also avoid *Bt* when laying eggs in a choice situation [38,39]. *Bt* avoidance was also reported in insects' offspring: neonates of the European corn borer*, Ostrinia nubilalis*, disperse more on *Bt* corn [40] and avoid *Bt* when facing a choice with untreated diet [41], while neonates of the tobacco budworm *Heliothis virescens* avoid diets containing Cry toxins or the *Bt* bioinsecticide at doses that do not alter their development and survival [42].

In non-target species, foraging activity and learning ability of *Apis mellifera ligustica* honeybees remained unchanged on *Bt* corn [43], while collective nest building and prey attacks were altered by cuticular *Bt* inoculation to the African social spider *Stegodyphus dumicola* [44]. Altered reproduction and survival were recorded in *Bombus terrestris* bumblebees exposed to *Bt* depending on the *Bt* subspecies and the exposure route, but without altering their foraging behaviour and colony performance [45]. *Bt* bioinsecticides being increasingly applied in the field, studies exploring the behavioural avoidance by non-target invertebrates are needed for an accurate assessment of the potential bioinsecticide side-effects.

Here, we explored the expression of behavioural avoidance toward the lepidopteran-targeting *Bt* var. *kurstaki* (*Btk*) bioinsecticide by non-target *Drosophila* species that exhibit developmental and physiological alterations in the chronic presence of bioinsecticide [27,28,46]. *Drosophila* larvae are particularly exposed to food-borne stressors as they intensively search for food to fuel their exponential growth but have a low dispersal capacity. Bioinsecticide avoidance by adult females when searching for oviposition sites would mitigate the consequences on larval development. We focused on three *Drosophila* species with different ecological features and varying developmental alterations elicited by chronic *Btk* exposure: two cosmopolitan domestic species which frequently coexist on overripe fruits, *D. melanogaster* (four strains) and the phylogenetically distant and opportunistic *D. busckii* [47–51], and the invasive *D. suzukii* that feeds and lays eggs on ripe fruits and is a threat to agriculture [52–55]. We measured the females’ oviposition preference in two-choice tests where they were offered food with or without *Btk* bioinsecticide at a specific dose. The preference dynamics during the choice test was recorded and the effect of different fractions of the *Btk* bioinsecticide suspension (spores and toxin crystals, and soluble toxins/compounds) on the fly preference was also assessed.

## 2. Material and methods

### 2.1. Fly stocks

Four strains of the model species *D. melanogaster* were tested: the wild-type Canton-S (Bloomington Drosophila Center) used here as a reference strain, the wild-type ‘Nasrallah’ from Tunisia (strain 1333, Gif-sur-Yvette), a wild-type strain ‘Sefra’ derived from flies collected in southern France in 2013, and the *yellow-white* double mutant *yw*^1118^ (gift from Dr B. Charroux, IBD, Marseille-Luminy). Those strains and the two other *Drosophila* species tested, *D. busckii* (derived from flies collected in southeast France in 2015) and *D. suzukii* (gift from Dr R. Allemand, LBBE, University of Lyon 1; originating from flies collected near Lyon), were reared under controlled laboratory conditions (150–200 eggs/40 ml fly medium; 25°C for *D. melanogaster* and 20°C for the two other fly species; 60% relative humidity; 12 : 12 light/dark cycle) on a high-protein/sugar-free fly medium (10% cornmeal, 10% yeast, 0% sugar). All the experiments were performed under these laboratory conditions.

### 2.2. *Bacillus thuringiensis* bioinsecticide product

Spores and Cry toxins of *Bt.* var. *kurstaki* strain SA-11 were from a commercial bioinsecticide product (Delfin wettable granules, Valent BioSciences, AMM 9200482, 32 000 IU mg^−1^). Viable spores were estimated at 5 × 10^7^ CFU mg^−1^ product by counting colony forming units (CFUs) on LB agar, and this value remained stable during the timeframe of this study. For the experiments, suspensions of *Btk* bioinsecticide were prepared in Ringer buffer (NaCl 7.5 g l^−1^, NaHCO_3_ 0.1 g l^−1^, KCl 0.2 g l^−1^, CaCl_2_ 0.2 g l^−1^, in distilled water) to reach the desired CFUs in 100 µl.

### 2.3. Oviposition choice test

Two-to-five day-old mated females (20 *D. melanogaster*, 30 *D. suzukii*, 30 *D. busckii*) were transferred to aerated plastic cages (Ø 10.5 cm, height 7.5 cm) containing two dishes (Ø 3 cm, approx. 7 cm^2^, 1 g of fly medium) diametrically opposed at the cage bottom. The test lasted 18 h for *D. melanogaster*, and 24 h for *D. suzukii* and *D. busckii* which lay fewer eggs per day. To avoid confounding effects, cage orientation and location in the experimental chamber were randomized.

### 2.4. Oviposition in presence of *Btk* bioinsecticide

Flies were given the choice between a dish filled with fly medium mixed with a suspension of *Btk* bioinsecticide in Ringer buffer at a given concentration, and a control dish filled with fly medium mixed with the same volume of Ringer buffer (dose ‘0’). In control cages, females were offered the choice between two dishes filled with fly medium mixed with Ringer buffer. Oviposition preference for *Btk* was calculated as the number of eggs laid on the *Btk* substrate divided by the total of eggs counted on the two substrates of the cage. Oviposition preference of 0.5 indicates neither preference nor avoidance of the bioinsecticide; oviposition preference values above 0.5 indicate bioinsecticide appetitiveness, while values below 0.5 indicate bioinsecticide avoidance. Oviposition preference in control cages was the egg proportion on one of the two Ringer substrates.

Three *Btk* bioinsecticide doses previously described in [27] were used: 10^6^ CFU g^−1^ fly medium that has no effect on the *Drosophila* development and falls in the recommendation range (equivalent to the field application of 1.4 × 10^5^ CFU cm^−2^) and 10^8^ and 10^9^ CFU g^−1^ which strongly alters *Drosophila* larval development (equivalent to the application of 1.4 × 10^7^ and 1.4 × 10^8^ CFU cm^−2^, respectively). The dynamics of egg laying over the 18 h choice test were explored with the *D. melanogaster* Canton-S strain by measuring the oviposition preference at 2, 4 and 18 h (endpoint) of choice test. Oviposition preference of *D. suzukii* and *D. busckii* was measured as the female choice over 24 h between a Ringer control substrate and a substrate containing 10^9^ CFU g^−1^ of *Btk* bioinsecticide.

To disentangle the effects on the oviposition preference of *Btk* spores, toxins (crystals and soluble toxins), and the commercial product additives, a 2 × 10^10^ CFU suspension of the bioinsecticide product was dialysed to remove low molecular weight compounds (such as additives) [27]. A fraction of the dialysed suspension was centrifuged at 15 000*g*, 15 min, 18°C to collect the pellet containing mainly spores and toxin crystals, and the supernatant containing toxin fragments and non-dialysable compounds [27]. The oviposition preference and total numbers of eggs laid of *D. melanogaster* Canton-S females were assessed during 18 h when flies were offered the choice between a control Ringer substrate and a substrate containing the non-dialysed bioinsecticide, the dialysed bioinsecticide, the centrifugation pellet (all adjusted to 10^9^ CFU g^−1^), the supernatant, or the PBS buffer used for dialysis.

### 2.5. Statistical analysis

Binomial data on oviposition preference were analysed with mixed-effects generalized linear models that included, when appropriate, the *D. melanogaster* strain, the *Btk* treatment (Ringer control, *Btk* bioinsecticide doses, dialysis and centrifugation fractions), the choice test duration and their two-way interactions as fixed factors. The replicate cage was included as random factor. Total numbers of eggs laid (counts) were transformed into decimal logarithm values and analysed with mixed-effect models including the same fixed and random effects as described above (similar statistical results and biological conclusions were obtained with untransformed data). Significance of fixed effects and interactions was tested by model comparisons with log-likelihood ratio tests. Pairwise *post hoc* comparisons of each *Btk* dose with the no-*Btk* control and of each fly strain with the standard strain Canton-S were performed. The deviation of the oviposition preference from a 50%−50% distribution of eggs on the two substrates was tested with *t*-tests under the H0 hypothesis of a mean egg proportion of 0.5. The replicate number being relatively small, Wilcoxon tests with the same H0 hypothesis were performed and yielded similar biological conclusions. Statistical analyses were performed in R [56] using the packages lme4 [57] and multcomp [58].

## 3. Results

### 3.1. *Drosophila melanogaster* expressed a rapid, dose-dependent oviposition avoidance of *Btk* bioinsecticide

**Figure 1.**
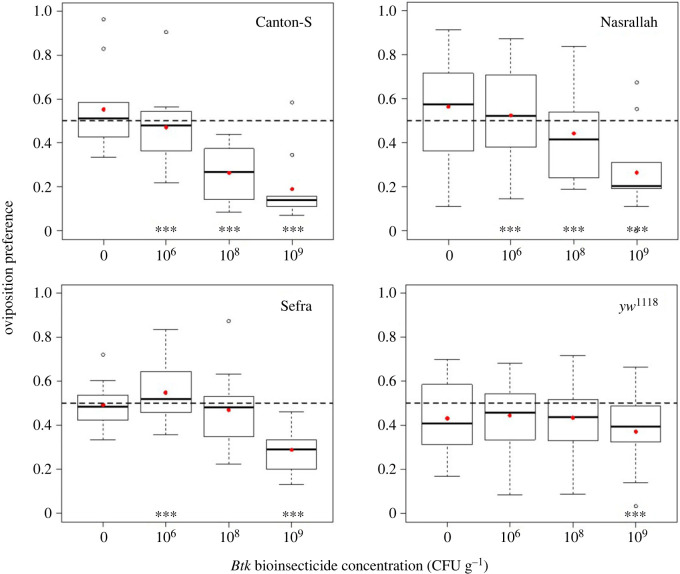
Female oviposition preference in the 18 h choice test as the proportion of eggs laid on one food substrate (quartiles, median and mean preference in red points) of *D. melanogaster* wild-type strains Canton-S, Nasrallah and Sefra, and the double mutant strain *yw*^1118^, with three doses of *Btk* bioinsecticide (10^6^, 10^8^, and 10^9^ CFU g^−1^ of fly medium) and the no-*Btk* Ringer control (0). Significance of *post hoc* pairwise comparisons of the control with each *Btk* dose: *** *p* < 0.0001. *N* = 10 replicate cages per treatment for each fly strain.

**Figure 2.**
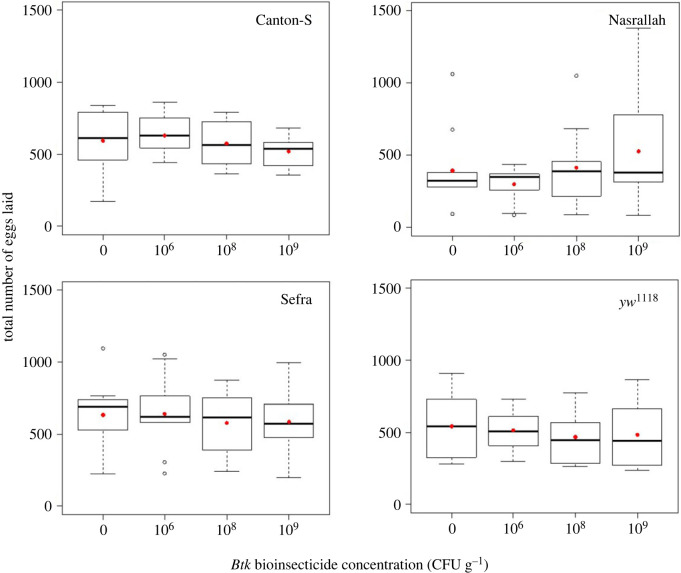
Total number of eggs laid on both food substrates offered during the 18 h oviposition choice test (quartiles, median and mean of the total number of eggs in red points) of *D. melanogaster* wild-type strains Canton-S, Nasrallah and Sefra, and the double mutant strain *yw*^1118^, with three doses of *Btk* bioinsecticide (10^6^, 10^8^ and 10^9^ CFU g^−1^ of fly medium) and the no-*Btk* Ringer control (0). *N* = 10 replicate cages per treatment for each fly strain.

The presence of *Btk* bioinsecticide impacted the oviposition preference of *D. melanogaster* females over 18 h compared with the controls without bioinsecticide, yet with varying amplitudes between fly strains (figure 1; electronic supplementary material, table S1.1). Canton-S females laid eggs evenly when offered the choice between two control substrates, while they laid fewer eggs on *Btk* substrate when offered a choice between substrates with and without *Btk* (electronic supplementary material, table S1.1; significance of *post hoc* control–*Btk* dose pairwise comparisons in figure 1). The *Btk* avoidance increased with the bioinsecticide dose, and deviated significantly from the ‘neutral’ preference of 0.5 at the two highest doses, 10^8^ and 10^9^ CFU g^−1^ (electronic supplementary material, table S1.1), dropping to 0.19 on average at 10^9^ CFU g^−1^ (95% CI: 0.07–0.30). The oviposition preference of Nasrallah females also decreased with the increasing *Btk* dose (figure 1; electronic supplementary material, table S1.1), dropping significantly below 0.5 only at 10^9^ CFU g^−1^ with a smaller amplitude than that of Canton-S females (0.27 on average, 95% CI: 0.12–0.41; electronic supplementary material, table S1.1). Similarly, the average preference of Sefra females was 0.29 at this dose (95% CI: 0.21–0.37), while the dose 10^6^ CFU g^−1^ was slightly appetitive (figure 1; electronic supplementary material, table S1.1). The oviposition preference of the double mutant *yw*^1118^ also decreased significantly below 0.5 at 10^9^ CFU g^−1^ but with smaller amplitude (average preference of 0.37, 95% CI: 0.24–0.50) (figure 1; electronic supplementary material, table S1.1). For all the four *D. melanogaster* strains, the total numbers of eggs laid during the course of the *Btk* choice tests were similar to those laid in control conditions, and similar between *Btk* doses (figure 2; electronic supplementary material, table S1.1).

**Figure 3.**
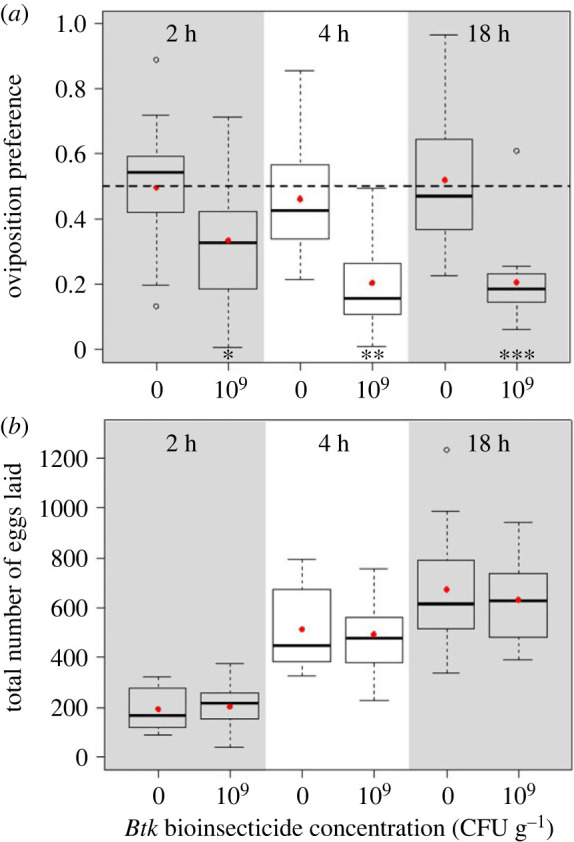
Dynamics of *D. melanogaster* Canton-S female (*a*) oviposition preference as the proportion of eggs laid on one food substrate, and (*b*) total number of eggs laid on both food substrates (quartiles, median and mean per treatment in red points) recorded at 2, 4 and 18 h in the oviposition choice test with 10^9^ CFU g^−1^ of *Btk* bioinsecticide and the no-*Btk* Ringer control (0). Significance of *post hoc* pairwise comparisons of the control with the *Btk* bioinsecticide: * *p* < 0.05, ** *p* < 0.01 *** *p* < 0.0001. *N* = 15 replicate cages per treatment and test duration.

Over the course of the 18 h choice test, the oviposition preference of the Canton-S females in control conditions did not differ from the ‘neutral’ preference 0.5, despite random variation across time points. By contrast, when offered the choice between a *Btk* substrate at 10^9^ CFU g^−1^ and a control substrate, the female preference for *Btk* was already below 0.5 at 2 h and further decreased at 4 h to remain down to approximately 0.2 until the end of the choice test (figure 3*a*; electronic supplementary material, table S1.2). The total numbers of eggs laid by Canton-S females evolved similarly and regardless of the choice they were offered (figure 3*b*; electronic supplementary material, table S1.2).

### 3.2. All the *Btk* bioinsecticide fractions elicited the fly oviposition avoidance

**Figure 4.**
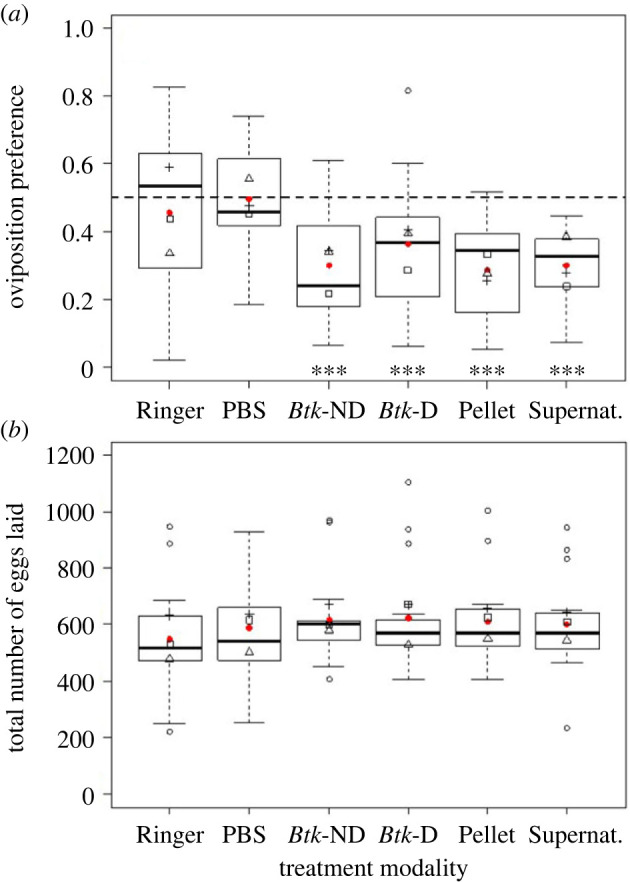
*Drosophila melanogaster* Canton-S female (*a*) oviposition preference as the proportion of eggs laid on one food substrate, and (*b*) total number of eggs laid on both food substrates (quartiles, median and mean per treatment in red points) in the 18 h oviposition choice test with *Btk* bioinsecticide at 10^9^ CFU g^−1^ of fly medium (*Btk-*ND), dialysed *Btk* bioinsecticide (*Btk*-D) and the pellet (Pellet) adjusted to the same concentration, the supernatant (Supernat.) after centrifugation, and the Ringer and PBS controls. Significance of *post hoc* pairwise comparisons of the Ringer control with each of the other treatment modalities: *** *p* < 0.001. *N* = 15 replicate cages per treatment.

While the preference after 18 h of Canton-S females for both Ringer and PBS controls did not differ from 0.5 (figure 4*a*, electronic supplementary material, table S2), females significantly avoided the dialysed *Btk* suspension, the suspended pellet and the supernatant with a similar amplitude to the non-dialysed *Btk* bioinsecticide at 10^9^ CFU g^−1^ (average preference of 0.30, 95% CI: 0.21–0.39; figure 4*a*, electronic supplementary material, table S2). The total number of eggs laid during the test were similar across choice modalities (figure 4*b*, electronic supplementary material, table S2).

### 3.3. The amplitude of fly avoidance of *Btk* bioinsecticide varied between species

**Figure 5.**
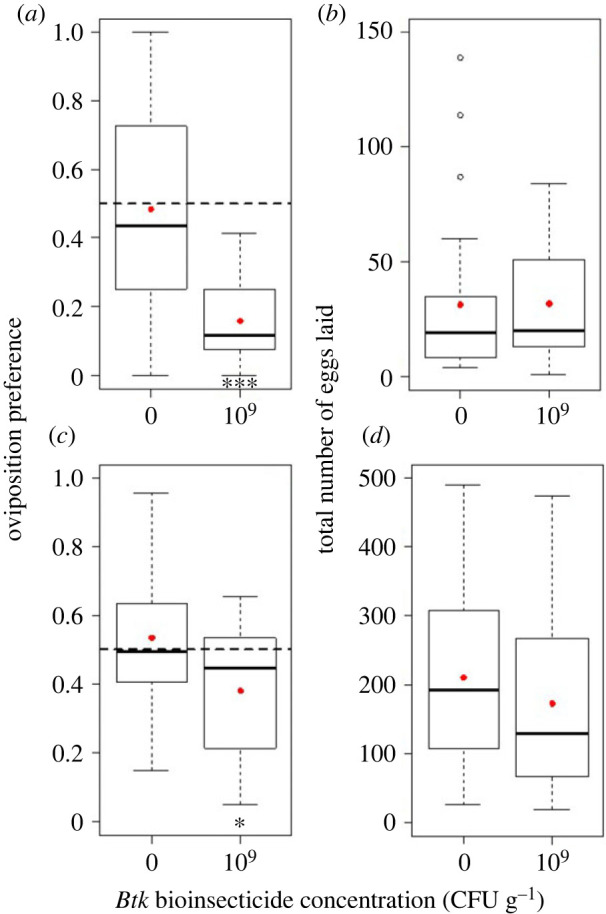
*Drosophila suzukii* (*a*) and *D. busckii* (*c*) female oviposition preference as the proportion of eggs laid on one food substrate, and (*b,d*) their respective total numbers of eggs laid on both food substrates during the 24 h oviposition choice test with *Btk* bioinsecticide at 10^9^ CFU g^−1^ and the no-*Btk* Ringer control (0) (quartiles, median and mean per treatment in red points). Significance of *post hoc* pairwise comparisons of the control with the *Btk* bioinsecticide: * *p* < 0.05 and *** *p* < 0.0001. *N* = 25 replicate cages per treatment for *D. suzukii* (all cages) and *N* = 15 replicate cages for *D. busckii*.

Females of the invasive species *D. suzukii* strongly avoided *Btk* in the choice test: their oviposition preference dropped to 0.16 on average in presence of 10^9^ CFU g^−1^ of *Btk* (95% CI: 0.11–0.21; figure 5*a*, electronic supplementary material, table S3), the results being similar when including only cages with more than 15 eggs laid (electronic supplementary material, figure S4). *Drosophila busckii* females' preference also dropped significantly to 0.38 on average in presence of 10^9^ CFU g^−1^ of *Btk* (95% CI: 0.28–0.49; figure 5*c*, electronic supplementary material, table S5). For the two fly species, the total numbers of eggs laid were independent of the choice offered (figures 5*b,d*; electronic supplementary material, figure S4; tables S3 and S5).

## 4. Discussion

When offered the choice between laying eggs on uncontaminated or *Btk-*contaminated substrates, females of *D. melanogaster* (all the strains used), *D. busckii* and *D. suzukii* expressed avoidance of the *Btk* bioinsecticide. The oviposition responses were independent of confounding differences in the total numbers of eggs laid during the choice test. Our study focusing on non-ageing mated females only, this also excludes the confounding effects of the female mating status and disturbance by male courtship, of sensory ageing impairing the ability to discriminate between food substrates, and of general ageing influencing the number of eggs laid.

The *D. melanogaster* strains avoided the bioinsecticide in a dose-dependent manner, and the three wild-type strains (Canton-S, Nasrallah, Sefra) showed a stronger avoidance of the highest bioinsecticide dose than the double mutant *yw*^1118^. The smaller avoidance amplitude by *yw*^1118^ females might coincide with the impacts of the yellow and white mutations on the flies’ non-social and social behaviour and their ability to learn with olfactory cues [59–62], yet this should be further explored with an appropriate genetic background control. The observed avoidance expressed by *D. melanogaster* was surprising given the fact that this fly species feeds on decaying organic matter (overripe fruits) probably full of microorganisms, which presence (detected by olfactory cues) enhances its egg laying [63]. The invasive Asian species, *D. suzukii*, exhibited a strong avoidance as the wild-type *D. melanogaster* Canton-S, although this species underwent an evolutionary shift in the bitter taste perception [64], but consistently with previous report of decreased egg laying in the presence of microorganisms [63]. The third species tested, *D. busckii* (subgenus *Dorsilopha*) of the *Drosophila* cosmopolitan guild of domestic species, feeds opportunistically on overripe fruits as *D. melanogaster* [47], and was the least avoidant species. This indicates the bioinsecticide avoidance was general to the species tested in our study, yet it occurred with inter-species variability.

*Drosophila melanogaster* bioinsecticide avoidance occurred as early as 2 h after the choice test onset, with increasing amplitude in the following few hours. This time scale is rapid on a fly lifetime's scale and consistent with previous reports of rapid learned avoidance towards pathogenic bacteria observed in *D. melanogaster* [12]*.* The bioinsecticide avoidance may have started earlier during the choice test, yet counting eggs laid does not provide a fine time resolution, since a robust result requires substantial numbers of eggs. Further video tracking method may help to investigate this. Nevertheless, it is known that the female decision-making for oviposition is a highly complex and dynamic trait that combines several parameters: the female's genotype and experience of the oviposition substrates [12,65–67], the presence at oviposition sites of the male-derived aggregation pheromone transmitted to females during mating and emitted by recently mated females and of the deterring host marking pheromone [68–70], the social transmission of oviposition substrate preferences between females [71–73] and of other information linked to substrate quality (presence of larvae and faeces) [70,74,75], the presence of specific commensal microorganisms [63,76], the amplification of pheromone aggregation signal in infected flies by pathogenic bacteria [77] and the group size [78]. The substrate texture also plays an important role in the female oviposition decisions [63,79]. In our study system, the bioinsecticide doses and Ringer buffer addition to the fly medium changed similarly the texture of the food substrate and did not change its pH [27].

All the fly strains and species avoided the bioinsecticide at the highest dose tested, 10^9^ CFU g^−1^ of *Btk*, and at 10^8^ CFU g^−1^ for *D. melanogaster* Canton-S and Nasrallah. This is in line with recent findings of development alterations upon chronic exposure to these doses, and the smaller bioinsecticide impacts on the emergence rates of *D. melanogaster yw*^1118^ and *D. busckii* compared with the other *D. melanogaster* strains and *D. suzukii* [27]. While the dose 10^9^ CFU g^−1^ is 1000 times above the manufacturer's recommendations and seems unrealistic in the field, the dose 10^8^ CFU g^−1^ (equivalent to a field application of 1.4 × 10^7^ CFU cm^−^^2^) [27] is reachable under current agricultural practices (up to eight authorized repeated applications are recommended [80,81] www.certiseurope.fr; www.certisusa.com). Indeed, *Bt* spores and toxins naturally persist and could accumulate [16,23,24,82], and bioinsecticide products contain protective compounds that lengthen their activity after field application [80,83]. Very recently, doses close to 10^8^ CFU g^−1^ were measured in honeybee matrices and flowers after field application of the maximum recommended *Bt* bioinsecticide dose, and concentrations up to 10^7^ CFU g^−1^ still persisted two days later [84].

Behavioural avoidance of *Btk* bioinsecticide also occurred with the dialysed suspension and each of its fractions independently. This excludes a main role of small molecular weight compounds present in the commercial product [85], and suggests the contribution of spores, toxins, or residual bacterial fragments in oviposition avoidance. Since *Bt* spores persist longer in the field than toxins [16,23,24], our results suggest that *Bt* spores in the environment may be sufficient to elicit oviposition avoidance by non-target *Drosophila* females. It is further known than the presence of bacterial cell wall components (possibly remaining after bioinsecticide manufacturing) induce bacteria avoidance in nematodes [86] and *D. melanogaster* males and females [14], which could be evaluated in our type of study system. At the mechanistic level, larvae and adult *Drosophila* naturally avoid specific harmful compounds or nutritionally unsuitable food based on the sensory perception of olfactory cues [87–89], gustatory cues [64,90–92], or the physiological consequences of ingesting virulent bacteria [12]. In our study, it seems unlikely that female bioinsecticide avoidance for oviposition relies only on olfactory cues, as this would probably result in stronger oviposition avoidance early during the test. Yet the involvement of gustatory cues (e.g. bitter taste) and/or physiological consequences of ingesting *Btk* bioinsecticide remains to be assessed. Indeed, it was shown very recently that *Bt* endotoxins could activate the *Drosophila* innate immune system and disrupt their gut cellular and endocrine homeostasis [93,94].

For the females’ offspring, oviposition avoidance of *Btk* bioinsecticide alleviates the cost of developing under chronic bioinsecticide exposure. Indeed, the growth and gut physiology of *D. melanogaster* larvae is dramatically disturbed already at 5 × 10^7^ CFU g^−1^ of bioinsecticide [28]. Emergence rates of *D. melanogaster* strains on 10^8^ CFU g^−1^ of bioinsecticide dropped by up to 81% [27]. The development success was even null at 10^9^ CFU g^−1^ [27]. Avoidance of *Btk* bioinsecticide by females while searching for oviposition sites would thus increase their inclusive fitness, providing their progeny more chances to develop and reach the adult stage and reproduce. Given that *Drosophila* females both feed and lay eggs on food substrates, the avoidance of *Btk*-contaminated oviposition sites would also reduce the adult fly exposure to bioinsecticide, although adults are not severely impacted [27].

From an ecological point of view, varying avoidance amplitudes between *D. melanogaster* genotypes and *Drosophila* species may modify their competitive interactions in *Btk*-treated areas. Variations in avoidance strength have already been observed for carbon dioxide and other odorants indicating the stage of the fruit ripeness, reflecting the biological differences between *Drosophila* species specialized on overripe fruits (*D. melanogaster*, *D. yakuba*, *D. pseudobscura*, *D. virilis*) and *D. suzukii* specialized on ripening fruits [87,88]. In our study, smaller avoidance amplitude of *D. busckii,* combined with its lower developmental susceptibility to chronic bioinsecticide exposure [27] suggest that *Btk* applications might not dramatically affect the field presence of this species in the *Drosophila* community. By contrast, the strong developmental alterations of *D. suzukii* upon chronic exposure to bioinsecticide [26,27], combined with the strong female oviposition avoidance, suggest that developmental alterations could be alleviated by avoidance of *Btk-*treated areas. Despite the fact that *D. melanogaster* and *D. suzukii* have different niche specializations, their potential indirect interactions would be displaced mostly to *Btk*-untreated areas since both species show strong oviposition avoidance. The population dynamics of their natural enemies (predators and parasites) would also be indirectly impacted by these distribution changes, in addition to potential direct impacts [95]. Interestingly, our results further indicate that *Btk* bioinsecticide might be an effective repellent to *D. suzukii* in orchards and gardening, but not a population control agent as it comes with side effects for other non-target fly species.

In summary, females of several *Drosophila* species and genotypes expressed oviposition avoidance of food substrates contaminated with *Btk* bioinsecticide. The avoidance appeared rapidly after the onset of choice tests, for all the fractions of the bioinsecticide suspension, and was independent of female motivation for egg laying. Our study extends the assessment of *Btk* bioinsecticide chronic effects previously reported in multiple *Drosophila* species to behavioural aspects, and highlights the need for multi-component assessments (development, physiology, life history, behaviour) of the potential effects of bioinsecticides on non-target invertebrates.

## Data Availability

The data are provided in electronic supplementary material [96].

## References

[1] Moore J. 2002 Behavioral alterations and avoiding parasites. In Parasites and the behaviour of animals (eds RM May, PH Harvey), pp. 89-118. Oxford, UK: Oxford University Press.

[2] Siva-Jothy MT, Moret Y, Rolff J. 2005 Insect immunity: an evolutionary ecology perspective. Adv. Insect Physiol. **32**, 1-48. (10.1016/S0065-2806(05)32001-7)

[3] Schmid-Hempel P. 2011 Evolutionary parasitology: the integrated study of infections, immunology, ecology, and genetics, 1st edn. Oxford, UK: Oxford University Press.

[4] Pursall ER, Rolff J. 2011 Immune responses accelerate ageing: proof-of-principle in an insect model. PLoS ONE **5**, e19972. (10.1371/journal.pone.0019972) PMC3097213 21625631

[5] Curtis V, de Barra M, Aunger R. 2011 Disgust as an adaptive system for disease avoidance behaviour. Phil. Trans. R. Soc. B **366**, 389-401. (10.1098/rstb.2010.0117) 21199843 PMC3013466

[6] Darmaillacq AS, Dickel L, Chichery MP, Agin V, Chichery R. 2004 Rapid taste aversion learning in adult cuttlefish, *Sepia officinalis*. Anim. Behav. **68**, 1291-1298. (10.1016/j.anbehav.2004.01.015)

[7] Wright GA, Mustard JA, Simcock NK, Ross-Taylor AAR, McNicholas LD, Popescu A, Marion-Poll F. 2010 Parallel reinforcement pathways for conditioned food aversions in the honeybee. Curr. Biol. **20**, 2234-2240. (10.1016/j.cub.2010.11.040) 21129969 PMC3011020

[8] Sellier MJ, Reeb P, Marion-Poll F. 2011 Consumption of bitter alkaloids in *Drosophila melanogaster* in multiple-choice test conditions. Chem. Sens. **36**, 323-334. (10.1093/chemse/bjq133) 21173029

[9] Papaj DR, Prokopy RJ. 1989 Ecological and evolutionary aspects of learning in phytophagous insects. Annu. Rev. Entomol. **34**, 315-350. (10.1146/annurev.en.34.010189.001531)

[10] Sicard M, Hering S, Schulte R, Gaudriault S, Schulenburg H. 2007 The effect of *Photorhabdus luminescens* (Enterobacteriaceae) on the survival, development, reproduction and behaviour of *Caenorhabditis elegans* (Nematoda: Rhabditidae). Environ. Microbiol. **9**, 12-25. (10.1111/j.1462-2920.2006.01099.x) 17227408

[11] Abada EA, Sung H, Dwivedi M, Park BJ, Lee SK, Ahnn J. 2009 *C. elegans* behavior of preference choice on bacterial food. Mol. Cells **28**, 209-213. (10.1007/s10059-009-0124-x) 19756391

[12] Babin A, Kolly S, Schneider F, Dolivo V, Zini M, Kawecki TJ. 2014 Fruit flies learn to avoid odours associated with virulent infection. Biol. Lett. **10**, 20140048. (10.1098/rsbl.2014.0048) 24598110 PMC3982440

[13] Zhang Y, Lu H, Bargmann CI. 2005 Pathogenic bacteria induce aversive olfactory learning in *Caenorhabditis elegans*. Nature **438**, 179-184. (10.1038/nature04216) 16281027

[14] Soldano A et al. 2016 Gustatory-mediated avoidance of bacterial lipopolysaccharides via TRPA1 activation in *Drosophila*. Elife **5**, e13133. (10.7554/eLife.13133) 27296646 PMC4907694

[15] Crickmore N. 2017 *Bacillus thuringiensis* toxin classification. In Bacillus thuringiensis and lysinibacillus sphaericus (eds LM Fiuza, RA Polanczyk, N Crickmore). Cham, Switzerland: Springer.

[16] Enger KS, Mitchell J, Murali B, Bridsell DN, Keim P, Gurian PL, Wagner DM. 2018 Evaluating the long-term persistence of *Bacillus* spores on common surfaces. Microb. Biotechnol. **11**, 1048-1059. (10.1111/1751-7915.13267) 29726106 PMC6196380

[17] Bravo A, Likitvivatanavong S, Gill SS, Soberon M. 2011 *Bacillus thuringiensis*: a story of a successful bioinsecticide. Insect Biochem. Mol. Biol. **41**, 423-431. (10.1016/j.ibmb.2011.02.006) 21376122 PMC3689885

[18] Sanchis V, Bourguet D. 2008 *Bacillus thuringiensis*: applications in agriculture and insect resistance management: a review. Agron. Sustain. Dev. **28**, 11-20. (10.1051/agro:2007054)

[19] Lacey LA, Grzywacz D, Shapiro-Ilan DI, Frutos R, Brownbridge M, Goettel MS. 2015 Insect pathogens as biological control agents: back to the future. J. Invertebr. Pathol. **132**, 1-41. (10.1016/j.jip.2015.07.009) 26225455

[20] Glare TR, O'Callaghan M. 2000 Bacillus thuringiensis: biology, ecology and safety. Chichester, UK: John Wiley & Sons.

[21] Rubio-Infante N, Moreno-Fierros L. 2016 An overview of the safety and biological effects of *Bacillus thuringiensis* Cry toxins in mammals. J. Appl. Toxicol. **36**, 630-648. (10.1002/jat.3252) 26537666

[22] EFSA Panel on Biological Hazards (BIOHAZ). 2016 Risks for public health related to the presence of *Bacillus cereus* and other *Bacillus* spp. including *Bacillus thuringiensis* in foodstuffs. EFSA J. **14**, e04524. (10.2903/j.efsa.2016.4524)

[23] Hung TP, Truong LV, Binh ND, Frutos R, Quiquampoix H, Staunton S. 2016 Persistence of detectable insecticidal proteins from *Bacillus thuringiensis* (Cry) and toxicity after adsorption on contrasting soils. Environ. Pollut. **208**, 318-325. (10.1016/j.envpol.2015.09.046) 26549751

[24] Hung TP, Truong LV, Binh ND, Frutos R, Quiquampoix H, Staunton S. 2016 Fate of insecticidal *Bacillus thuringiensis* Cry protein in soil: differences between purified toxin and biopesticide formulation. Pest Manag. Sci. **72**, 2247-2253. (10.1002/ps.4262) 26910634

[25] van Frankenhuyzen K. 2017 Specificity and cross-order activity of *Bacillus thuringiensis* pesticidal proteins. In Bacillus thuringiensis and lysinibacillus sphaericus (eds LM Fiuza, RA Polanczyk, N Crickmore), pp. 127-172. Cham, Switzerland: Springer.

[26] Cossentine J, Robertson M, Xu D. 2016 Biological activity of *Bacillus thuringiensis* in *Drosophila suzukii* (Diptera: Drosophilidae). J. Econ. Entomol. **109**, 1-8. (10.1093/jee/tow062) 27106227

[27] Babin A, Nawrot-Esposito M-P, Gallet A, Gatti J-L, Poirié M. 2020 Differential side-effects of *Bacillus thuringiensis* bioinsecticide on non-target *Drosophila* flies. Sci. Rep. **10**, 16241. (10.1038/s41598-020-73145-6) 33004867 PMC7529784

[28] Nawrot-Esposito M-P, Babin A, Pasco M, Poirié M, Gatti J-L, Gallet A. 2020 *Bacillus thuringiensis* bioinsecticides induce developmental defects in non-target *Drosophila melanogaster* larvae. Insects **11**, 697. (10.3390/insects11100697) 33066180 PMC7601982

[29] Mastore M, Quadroni S, Brivio MF. 2021 Susceptibility of *Drosophila suzukii* larvae to the combined administration of the entomopathogens *Bacillus thuringiensis* and *Steinernema carpocapsae*. Sci. Rep. **11**, 8149. (10.1038/s41598-021-87469-4) 33854098 PMC8046782

[30] Bellile KG, Vonesh JR. 2016 Bioinsecticide and leaf litter combination increases oviposition and reduces adult recruitment to create an effective ovitrap for *Culex* mosquitoes. J. Vector Ecol. **41**, 122-126. (10.1111/jvec.12203) 27232134

[31] Petzold-Maxwell JL, Cibilis-Stewart X, Wade French B, Gassmann AJ. 2012 Adaptation by western corn rootworm (Coleoptera: Chrysomelidae) to *Bt* maize: inheritance, fitness costs, and feeding preference. J. Econ. Entomol. **105**, 1407-1418. (10.1603/EC11425) 22928323

[32] Tellez-Rodriguez P, Raymond B, Moran-Bertot I, Rodriguez-Cabrera L, Wright DJ, Borroto CG, Ayra-Pardo C. 2014 Strong oviposition preference for *Bt* over non-Bt maize in *Spodoptera frugiperda* and its implications for the evolution of resistance. BMC Biol. **12**, 48. (10.1186/1741-7007-12-48) 24935031 PMC4094916

[33] Schulenburg H, Müller S. 2004 Natural variation in the response of *Caenorhabditis elegans* towards *Bacillus thuringiensis*. Parasitology **128**, 433-443. (10.1017/S003118200300461X) 15151149

[34] Hasshoff M, Boehnisch C, Tonn D, Hasert B, Schulenburg H. 2007 The role of *Caenorhabditis elegans* insulin-like signalling in the behavioural avoidance of pathogenic *Bacillus thuringiensis*. FASEB J. **21**, 1801-1812. (10.1096/fj.06-6551com) 17314144

[35] Schulte RD, Hasert B, Makus C, Michiels NK, Schulenburg H. 2012 Increased responsiveness in feeding behaviour of *Caenorhabditis elegans* after experimental coevolution with its microparasite *Bacillus thuringiensis*. Biol. Lett. **8**, 234-236. (10.1098/rsbl.2011.0684) 21880622 PMC3297370

[36] Wang J, Peng YD, He C, Wei BW, Liang YS, Yang HL, Wang Z, Stanley D, Song QS. 2012 Cry1Ab-expressing rice did not influence expression of fecundity-related genes in the wolf spider *Pardosa pseudoannulata*. Gene **592**, 1-7. (10.1016/j.gene.2016.07.041) 27452121

[37] Singh G, Rup PJ, Koul O. 2008 Selective feeding of *Helicoverpa armigera* (Hübner) and *Spodoptera litura* (Fabricius) on meridic diet with *Bacillus thuringiensis* toxins. J. Insect Behav. **21**, 407-421. (10.1007/s10905-008-9139-y)

[38] Zago HB, Siqueira HAA, Pereira EJG, Picanço MC, Barros R. 2014 Resistance and behavioural response of *Plutella xylostella* (Lepidoptera: Plutellidae) populations to *Bacillus thuringiensis* formulations. Pest Manag. Sci. **70**, 488-495. (10.1002/ps.3600) 23813721

[39] Zhao D, Zalucki MP, Guo R, Fang Z, Shen W, Zhang L, Liu B. 2016 Oviposition and feeding avoidance in *Helicoverpa armigera* (Hübner) against transgenic Bt cotton. J. Appl. Entomol. **140**, 715-724. (10.111/jen.12304)

[40] Razze JM, Mason CE. 2012 Dispersal behavior of neonate European corn borer (Lepidoptera: Crambidae) on Bt corn. J. Econ. Entomol. **105**, 1214-1223. (10.1603/EC11288) 22928300

[41] Girón-Calva PS, Loopez C, Albacete A, Albajes R, Christou P, Eizaguirre M. 2001 β-carotene and *Bacillus thuringiensis* insecticidal protein differentially modulate feeding behaviour, mortality and physiology of European corn borer (*Ostrinia nubilalis*). PLoS ONE **16**, e0246696. (10.1371/journal.pone.0246696) PMC7886157 33591990

[42] Gould F, Anderson A, Landis D, Van Mellaert H. 1991 Feeding behavior and growth of *Heliothis virescens* larvae on diets containing *Bacillus thuringiensis* formulations or endotoxins. Entomol. Exp. Appl. **58**, 199-210. (10.1111/j.1570-7458.1991.tb01469.x)

[43] Dai P-L, Zhou W, Zhang J, Cui H-J, Wang Q, Jiang W-Y, Sun J-H, Wu Y-Y, Zhou T. 2012 Field assessment of Bt *cry1Ah* corn pollen on the survival, development and behavior of *Apis mellifera ligustica*. Ecotox. Environ. Safe. **79**, 232-237. (10.1016/j.ecoenv.2012.01.005) 22364780

[44] Keiser CN, Wright CM, Pruitt JN. 2016 Increased bacterial load can reduce or negate the effects pf keystone individuals on group collective behaviour. Anim. Behav. **114**, 211-218. (10.1016/j.anbehav.2016.02.010)

[45] Mommaerts V, Jans K, Smagghe G. 2010 Impact of *Bacillus thuringiensis* strains on survival, reproduction and foraging behaviour in bumblebees (*Bombus terrestris*). Pest Manag. Sci. **66**, 520-525. (10.1002/ps.1902) 20024947

[46] Babin A, Gatti J-L, Poirié M. In press. *Bacillus thuringiensis* bioinsecticide influences *Drosophila* oviposition decision. BioRxiv. (10.1101/2023.03.07.531532) PMC10465210 37650056

[47] Atkinson W, Shorrocks B. 1977 Breeding site specificity in the domestic species of *Drosophila*. Oecologia **29**, 223-232. (10.1007/BF00345697) 28309117

[48] Shorrocks B. 1991 Competition on a divided and ephemeral resource: a cage experiment. Biol. J. Linn. Soc. **43**, 211-220. (10.1111/j.1095-8312.1991.tb00594.x)

[49] Benado M, Brncic D. 1994 An eight-year phenological study of a local drosophilid community in Central Chile. J. Zool. Syst. Evol. Res. **32**, 51-63. (10.1111/j.1439-0469.1994.tb00470.x)

[50] Nunney L. 1996 The colonization of oranges by the cosmopolitan *Drosophila*. Oecologia **108**, 552-561. (10.1007/BF00333733) 28307873

[51] Mitsui H, Kimura MT. 2000 Coexistence of drosophilid flies: aggregation, patch size diversity and parasitism. Ecol. Res. **15**, 93-100. (10.1046/j.1440-1703.2000.00328.x)

[52] Walsh DB et al. 2011 *Drosophila suzukii* (Diptera: Drosophilidae): invasive pest of ripening soft fruit expanding its geographic range and damage potential. J. Integr. Pest Manag. **2**, G1-G7. (10.1603/IPM10010)

[53] Delbac L, Rusch A, Rouzes R, Ravidat M-L, Launes S, Thiéry D. 2014 *Drosophila suzukii* est elle une menace pour la vigne? Phytoma **679**, 16-21.

[54] Poyet M, Eslin P, Héraude M, Le Roux V, Prévost G, Gibert P, Chabrerie O. 2014 Invasive host for invasive pest: when the Asiatic cherry fly (*Drosophila suzukii*) meets the American black cherry (*Prunus serotine*) in Europe. Agric. For. Entomol. **16**, 251-259. (10.1111/afe.12052)

[55] Tait G et al. 2021 *Drosophila suzukii* (Diptera: Drosophilidae): a decade of research towards a sustainable integrated pest management program. J. Econ. Entomol. **114**, 1950-1974. (10.1093/jee/toab158) 34516634

[56] R Core Team. 2008 R: A language and environment for statistical computing. Vienna, Austria: R Foundation for Statistical Computing.

[57] Bates D, Maechler M, Bolker B, Walker S. 2015 Fitting linear mixed-effects models using lme4. J. Stat. Softw. **67**, 1-48. (10.18637/jss.v067.i01)

[58] Hothorn T, Bretz F, Westfall P. 2008 Simultaneous inference in general parametric models. Biometrical J. **50**, 346-363. (10.1002/bimj.200810425) 18481363

[59] Bastock M. 1956 A gene mutation which changes a behavior pattern. Evolution **10**, 421-439. (10.2307/2407002)

[60] Anaka M, MacDonald CD, Barkova E, Simon K, Rostom R, Godoy RA, Haigh AJ, Meinertzhagen IA, Lloyd V. 2008 The *white* gene of *Drosophila melanogaster* encodes a protein with a role in courtship behaviour. J. Neurogenetics **22**, 243-276. (10.1080/01677060802309629) 19012054

[61] Simon AF, Chou M-T, Salazar ED, Nicholson T, Saini N, Metchev S, Krantz DE. 2012 A simple assay to study social behavior in *Drosophila*: measurement of social space within a group. Genes Brain Behav. **11**, 243-252. (10.1111/j.1601-183X.2011.00740.x) 22010812 PMC3268943

[62] Myers JL, Porter M, Narwold N, Bhat K, Dauwalder B, Roman G. 2021 Mutants of the *white* ABCG transporter in *Drosophila melanogaster* have deficient olfactory learning and cholesterol homeostasis. Int. J. Mol. Sci. **22**, 12967. (10.3390/ijms222312967) 34884779 PMC8657504

[63] Sato A, Tanaka KM, Yew JY, Takahashi A. 2021 *Drosophila suzukii* avoidance of microbes in oviposition choice. R. Soc. Open Sci. **8**, 201601. (10.1098/rsos.201601) 33614092 PMC7890486

[64] Dweck HKM, Talross GJS, Wang W, Carlson JR. 2021 Evolutionary shifts in taste coding in the fruit pest *Drosophila suzukii*. eLife **10**, e64317. (10.7554/eLife.64317) 33616529 PMC7899650

[65] Mery F, Kawecki TJ. 2002 Experimental evolution of learning ability in fruit flies. Proc. Natl Acad. Sci. USA **99**, 14 274-14 279. (10.1073/pnas.2223711) PMC137874 12391295

[66] Miller PM, Saltz JB, Cochrane VA, Marcinkowski CM, Mobin R, Turner TL. 2011 Natural variation in decision-making behavior in *Drosophila melanogaster*. PLoS ONE **6**, e16436. (10.1371/journal.pone.0016436) 21283727 PMC3024433

[67] McConnel MW, Fitzpatrick MJ. 2017 ‘Foraging’ for a place to lay eggs: a genetic link between foraging behaviour and oviposition preferences. PLoS ONE **12**, e0179362. (10.1371/journal.pone.0179362) 28622389 PMC5473555

[68] Bartelt RJ, Schaner AM, Jackson LL. 1985 *cis*-vaccenyl acetate as an aggregation pheromone in *Drosophila melanogaster*. J. Chem. Ecol. **11**, 1747-1756. (10.1007/BF01012124) 24311338

[69] Wertheim B, Allemand R, Vet LEM, Dicke M. 2006 Effects of aggregation pheromone on individual behaviour and food web interactions: a field study on *Drosophila*. Ecol. Entomol. **31**, 216-226. (10.1111/j.1365-2311.2006.00757.x)

[70] Elsensohn JE, Aly MFK, Schal C, Burrack HJ. 2021 Social signals mediate oviposition site selection in *Drosophila suzukii*. Sci. Rep. **11**, 3796. (10.1038/s41598-021-83354-2) 33589670 PMC7884846

[71] Sarin S, Dukas R. 2009 Social learning about egg-laying substrates in fruitflies. Proc. R. Soc. B **276**, 4323-4328. (10.1098/rspb.2009.1294) PMC2817106 19759037

[72] Battesti M, Moreno C, Joly D, Mery F. 2012 Spread of social information and dynamics of social transmission within *Drosophila* groups. Curr. Biol. **22**, 309-313. (10.1016/j.cub.2011.12.050) 22264604

[73] Battesti M, Moreno C, Joly D, Mery F. 2015 Biased social transmission in *Drosophila* oviposition choice. Behav. Ecol. Sociobiol. **69**, 83-87. (10.1007/s00265-014-1820-x)

[74] Durisko Z, Anderson B, Dukas R. 2014 Adult fruit fly attraction to larvae biases experience and mediates social learning. J. Exp. Biol. **217**, 1193-1197. (10.1242/jeb.097683) 24311811

[75] Keesey IW, Koerte S, Retzke T, Haverkamp A, Hansson BS, Knaden M. 2016 Adult frass provides a pheromone signature for *Drosophila* feeding and aggregation. J. Chem. Ecol. **42**, 739-747. (10.1007/s10886-016-0737-4) 27539589 PMC5045843

[76] Scheidler NH, Liu C, Hamby KA, Zalom FG, Syed Z. 2015 Volatile codes: correlation of olfactory signals and reception in *Drosophila*-yeast chemical communication. Sci. Rep. **5**, 14059. (10.1038/srep14059) 26391997 PMC4585764

[77] Keesey IW et al. 2017 Pathogenic bacteria enhance dispersal through alteration of *Drosophila* social communication. Nat. Commun. **8**, 265. (10.1038/s41467-017-00334-9) 28814724 PMC5559524

[78] Lihoreau M, Clarke IM, Buhl J, Sumpter DJT, Simpson SJ. 2016 Collective selection of food patches in *Drosophila*. J. Exp. Biol. **219**, 668-675. (10.1242/jeb.127431) 26747899

[79] Atkinson WD. 1983 Gregarious oviposition in *Drosophila melanogaster* is explained by surface texture. Aust. J. Zool. **31**, 925-929. (10.1071/ZO9830925)

[80] Brar SK, Verma M, Tyagi RD, Valéro JR. 2006 Recent advances in downstream processing and formulations of *Bacillus thuringiensis* based biopesticides. Process Biochem. **41**, 323-342. (10.1016/j.procbio.2005.07.015)

[81] European Food Safety Authority. 2012 Conclusion on the peer review of the pesticide risk assessment of the active substance *Bacillus thuringiensis* subsp. kurstaki (strains ABTS 351, PB 54, SA 11, SA 12, EG 234). EFSA J. **10**, 2540. (10.2903/j.efsa.2012.2540) PMC8535438 34721699

[82] Raymond B, Wyres KL, Sheppard SK, Ellis RJ, Bonsall MB. 2010 Environmental factors determining the epidemiology and population genetic structure of the *Bacillus cereus* group in the field. PLoS Pathog. **6**, e1000905. (10.1371/journal.ppat.1000905) 20502683 PMC2873914

[83] Couch TL. 2000 Industrial fermentation and formulation of entomopathogenic bacteria. In Entomopathogenic bacteria: from laboratory to field application (eds JF Charles, A Delécluse, NL Roux C), pp. 297-316. Dordrecht: Springer.

[84] Alkassab AT, Beims H, Janke M, Pistorius J. 2022 Determination, distribution, and environmental fate of *Bacillus thuringiensis* spores in various honeybee matrices after field application as plant protection product. Environ. Sci. Pollut. Res. Int. **17**, 25 995-26 001. (10.1007/s11356-022-19414-5) PMC8986675 35218483

[85] Knowles A. 2008 Recent developments of safer formulations of agrochemicals. Environmentalist **28**, 35-44. (10.1007/s10669-007-9045-4)

[86] Pradel E, Zhang Y, Pujol N, Matsuyama T, Bargmann CI, Ewbank JJ. 2007 Detection and avoidance of a natural product from the pathogenic bacterium *Serratia marcescens* by *Caenorhabditis elegans*. Proc. Natl Acad. Sci. USA **104**, 2295-2300. (10.1073/pnas.0610281104) 17267603 PMC1892944

[87] Pham CK, Ray A. 2015 Conservation of olfactory avoidance in *Drosophila* species and identification of repellents for *Drosophila suzukii*. Sci. Rep. **5**, 11527. (10.1038/srep11527) 26098542 PMC4476414

[88] Versace E, Eriksson A, Rocchi F, Castellan I, Sgado P, Albrecht H. 2016 Physiological and behavioral responses in *Drosophila melanogaster* to odorants present at different plant maturation stages. Physiol. Behav. **163**, 322-331. (10.1016/j.physbeh.2016.05.027) 27195459

[89] Wallingford AK, Hesler SP, Cha DH, Loeb GM. 2016 Behavioral response of spotted-wing drosophila, *Drosophila suzukii* Matsumura, to aversive odors and a potential oviposition deterrent in the field. Pest Manag. Sci. **72**, 701-706. (10.1002/ps.4040) 25973596

[90] König C, Schleyer M, Leibiger J, El-Keredy A, Gerber B. 2014 Bitter-sweet processing in larval *Drosophila*. Chem. Sens. **39**, 489-505. (10.1093/chemse/bju016) 24833133

[91] Poudel S, Lee Y. 2016 Gustatory receptors required for avoiding the toxic compound coumarin in *Drosophila melanogaster*. Mol. Cells **39**, 310-315. (10.14348/molcells.2016.2250) 26912085 PMC4844937

[92] Kaushik S, Kumar R, Kain P. 2018 Salt and essential nutrient: advances in understanding salt taste detection using *Drosophila* as a model system. J. Exp. Neurosci. **12**, 1-12. (10.1177/1179069518806894) PMC6249657 30479487

[93] Jneid R, Loudhaief R, Zucchini-Pascal N, Nawrot-Esposito MP, Fichant A, Rousset R, Bonis M, Osman D, Gallet A. 2023 *Bacillus thuringiensis* toxins divert progenitor cells toward enteroendocrine fate by decreasing cell adhesion with intestinal stem cells in *Drosophila*. Elife **12**, e80179. (10.7554/eLife.80179) 36847614 PMC9977296

[94] Mouawad C, Kallassy Awad M, Liegois S, Ferrandon D, Sanchis-Borja V, El Chamy L. 2023 The NF-κB factor Relish is essential for the epithelial defenses protecting against δ-endotoxin dependent effects of *Bacillus thuringiensis israelensis* infection in the *Drosophila* model. Res. Microbiol. **174**, 104089. (10.1016/j.resmic.2023.104089) 37348743

[95] Babin A, Lemauf S, Rebuf C, Poirié M, Gatti J-L. 2022 Effects of *Bacillus thuringiensis kurstaki* bioinsecticide on two non-target *Drosophila* larval endoparasitoid wasps. Entomol. Gen. **42**, 611-620. (10.1127/entomologia/2022/1452)

[96] Babin A, Gatti J-L, Poirié M. 2023 *Bacillus thuringiensis* bioinsecticide influences *Drosophila* oviposition decision. Figshare. (10.6084/m9.figshare.c.6794049) PMC10465210 37650056

